# Expression status of p53 and organic cation transporter 1 is correlated with poor response to preoperative chemotherapy in esophageal squamous cell carcinoma

**DOI:** 10.1186/s12957-022-02571-9

**Published:** 2022-04-01

**Authors:** Masahiro Izutsu, Takanori Domoto, Shingo Kamoshida, Hiroyuki Ohsaki, Hiroshi Matsuoka, Yusuke Umeki, Kazuya Shiogama, Masaya Hirayama, Koichi Suda, Ichiro Uyama

**Affiliations:** 1 Laboratory of Pathology, Department of Medical Biophysics, Kobe University Graduate School of Health Sciences, 7-10-2 Tomogaoka, Suma-ku, Kobe, Hyogo 654-0142 Japan; 2 Department of Surgery, Fujita Health University School of Medicine, 1-98 Dengakugakubo, Kutsukake-cho, Toyoake, Aichi 470-1192 Japan; 3 Department of Morphology and Cell Function, Fujita Health University School of Health Sciences, 1-98 Dengakugakubo, Kutsukake-cho, Toyoake, Aichi 470-1192 Japan

**Keywords:** Cisplatin, Esophageal squamous cell carcinoma, Preoperative chemotherapy, Organic cation transporter 1, p53

## Abstract

**Background:**

Esophageal squamous cell carcinoma (ESCC) is a highly malignant neoplasm. DNA-damaging drugs, such as cisplatin (CDDP) and 5-fluorouracil (5-FU), are most frequently used in preoperative chemotherapy for ESCC. However, the response to preoperative chemotherapy varies among patients. p53, encoded by *TP53*, participates in apoptotic pathways following chemotherapy with DNA-damaging drugs, and mutation of *TP53* contributes to chemoresistance. Organic cation transporter 1 (OCT1) participates in the uptake of CDDP, and its reduced expression is associated with CDDP resistance. The aim of this study was to evaluate the predictive impact of the expression status of p53 and OCT1 in response to preoperative chemotherapy in ESCC.

**Methods:**

We retrospectively assessed 66 ESCC patients who received preoperative chemotherapy with CDDP/5-FU (CF) or docetaxel/CDDP/5-FU (DCF). p53 and OCT1 expression in pretreatment biopsy specimens was immunohistochemically determined and correlated with histological response to preoperative chemotherapy.

**Results:**

p53 with wild-type (p53^WT-ex^) and mutant-type (p53^MT-ex^) expression patterns was identified in 40.9% and 59.1% of patients, respectively. High expression of OCT1 (OCT1^High^) was detected in 45.5%, and the remaining 54.5% showed low expression (OCT1^Low^). In a univariate analysis of the entire cohort, p53^MT-ex^ was significantly correlated with poor response (*P* = 0.026), whereas OCT1^Low^ showed marginal significance (*P* = 0.091). In a combined analysis, tumors with either p53^MT-ex^ or OCT1^Low^ showed a significant correlation with poor response compared with tumors with both p53^WT-ex^ and OCT1^High^ (*P* < 0.001). The sensitivity, specificity, and accuracy of combined p53/OCT1 were 93.9%, 47.1%, and 81.8%, respectively. Multivariate analysis identified p53 (*P* = 0.017), OCT1 (*P* = 0.032), and combined p53/OCT1 (*P* < 0.001) as independent predictors of histological response. When samples were stratified according to chemotherapy regimen in the univariate analysis, combined p53/OCT1 was the only significant factor for poor response in the CF (*P* = 0.011) and DCF (*P* = 0.021) groups, whereas p53 showed no statistical significance.

**Conclusions:**

Our results suggest that either p53^MT-ex^ or OCT1^Low^ expression in pretreatment biopsy specimens may be a potential predictor of poor response to preoperative chemotherapy with the CF-based regimens in ESCC, although the specificity needs to be improved.

## Background

Esophageal cancer is the seventh most common cancer and the sixth leading cause of cancer deaths worldwide [1]. Preoperative chemotherapy followed by surgery is an effective treatment for advanced esophageal squamous cell carcinoma (ESCC) [2]. Among several chemotherapy regimens, cisplatin (CDDP)/5-fluorouracil (5-FU) (CF) and docetaxel (DTX)/CDDP/5-FU (DCF) are promising regimens [3–5], and cytotoxic drugs remain the mainstay of treatment for ESCC. However, the therapeutic effects of preoperative chemotherapy vary among patients, and satisfactory treatment outcomes cannot be obtained in a proportion of patients [3–5]. Therefore, the identification of predictors of response to preoperative chemotherapy may help to avoid the administration of ineffective anticancer drugs that have unpleasant side effects as well as mitigate the risk of delaying surgery in patients who do not respond to preoperative chemotherapy. However, no such predictors have been established for ESCC.

p53, which is encoded by *TP53*, is a tumor suppressor that plays a pivotal role in regulating cell proliferation [6–8]. p53 is activated by cellular stress that leads to DNA damage, hypoxia, and oncogene activation and induces either cell-cycle arrest to allow DNA repair or apoptosis to remove the damaged cells. However, *TP53* is mutated in more than 50% of human cancers, including at least 40% of esophageal cancers [9–12], resulting in the disruption of its protective tumor-suppressor functions [7]. DNA-damaging drugs, such as CDDP and 5-FU, act by inducing DNA damage, which is a potent trigger for activation of *TP53* [6, 13, 14]. Therefore, functional inactivation of p53 by mutation could be associated with resistance to DNA-damaging chemotherapy.

The effectiveness of chemotherapy is also dependent on the intracellular accumulation of anticancer drugs. Cellular uptake of anticancer drugs is mediated by facilitated transport systems via solute carrier (SLC) transporters, suggesting that the expression of SLC transporters may help to predict the response to chemotherapy [15–18]. The organic cation transporters (OCTs) of the SLC22A family consist of three isoforms, OCT1, OCT2, and OCT3, which mediate the uptake transport of various organic cations including endogenous compounds, xenobiotics, and anticancer drugs [15–18]. An in vitro study demonstrated that transfection of OCT1 increased the accumulation and cytotoxicity of CDDP in human embryonic kidney cells [19], although another study presented results that were negative for the involvement of OCT1 in the accumulation and cytotoxicity of CDDP [20]. Furthermore, it was reported that *OCT1* silencing impaired the cytotoxic effects of CDDP in ESCC cells, and that the level of *OCT1* mRNA expression was downregulated in CDDP-resistant ESCC cells [21]. However, the clinical significance of OCT1 in ESCC, including its correlation with chemotherapeutic effects, remains to be elucidated.

Considering the above data, we speculated that a tumor-specific expression pattern of p53 and OCT1 could have a potential impact for predicting the effects of preoperative chemotherapy in ESCC. Therefore, we evaluated the expression status of p53 and OCT1 in the pretreatment biopsy specimens of patients with ESCC receiving preoperative chemotherapy and analyzed their correlation with response to preoperative chemotherapy.

## Methods

### Patients and tissue samples

This was a retrospective observational study. A total of 95 esophageal cancer patients with clinical stages (cStages) II to IVa were treated with preoperative chemotherapy followed by esophagectomy and regional lymph node dissection at Fujita Health University Hospital from 2013 to 2021. No patients received adjuvant therapy until relapse occurred. Four adenocarcinoma patients were excluded from the present study because critical molecular differences underlying ESCC and esophageal adenocarcinoma have been demonstrated in comprehensive genomic analyses [22]. Moreover, 25 patients were excluded because the tissue specimens were too small for immunohistochemical analysis, and thus, the remaining 66 ESCC patients were included in the present study.

Biopsy specimens with multiple samples (median, 3; range 2–7) obtained before preoperative chemotherapy (pretreatment biopsy specimens) and resection materials obtained after preoperative chemotherapy (posttreatment resection materials) were fixed in 10% neutral buffered formalin and embedded in paraffin. Three-micrometer-thick sections were cut, mounted on aminopropyltriethoxysilane-coated slides, and stained with hematoxylin and eosin (H&E) to assess the histological response to preoperative chemotherapy. All the patients provided written informed consent regarding the use of their tissue specimens, and this study was conducted with the approval of the ethics committees of Fujita Health University School of Medicine and Kobe University Graduate School of Health Sciences (reference numbers HM20-146 and 980, respectively).

### Chemotherapy regimen

All patients had a performance status of 0 or 1. Forty-seven patients with cStage II or III disease received the CF regimen, which consisted of CDDP 80 mg/m^2^ administered as a 2-h intravenous infusion on day 1 and 5-FU 800 mg/m^2^ as a 24-h continuous infusion on days 1–5 of a 21-day cycle. The remaining 19 patients with cStage III or IVa received the DCF regimen, which consisted of DTX 70 mg/m^2^ administered as a 1-h intravenous infusion on day 1, CDDP 70 mg/m^2^ as a 2-h intravenous infusion on day 1, and 5-FU 750 mg/m^2^ as a 24-h continuous infusion on days 1–5 of a 21-day cycle.

Two cycles of these regimens were administered. For patients who had or seemed to have adjacent organ invasion, two additional cycles were performed depending on their responsiveness to preoperative chemotherapy and the resectability of their tumor. Surgery was conducted approximately 4–6 weeks after the end of preoperative chemotherapy.

### Assessment of tumor response to preoperative chemotherapy

Histological response to preoperative chemotherapy was evaluated according to the proportion of viable cells in whole tumor tissue on H&E-stained sections of the posttreatment resection materials, according to the Japanese Classification of Esophageal Cancer, 11th edition [23], as follows: grade 0, no effect (no therapeutic effect is recognized in cancer cells and tissues); grade 1, slight effect (grade 1a, viable cancer cells occupy two-thirds or more of the entire tumor area; grade 1b, viable cancer cells remain in more than one-third but less than two-thirds of the entire tumor area); grade 2, considerable effect (viable cancer cells remain in less than one-third of the entire tumor area); and grade 3, complete response (no viable cancer cells remain). The response of tumors with grades 1b, 2, and 3 was judged as a “good response,” and that of tumors with grades 0 and 1a as a “poor response,” in accordance with previous studies [24].

### Immunostaining

Sections were deparaffinized in xylene, rehydrated in graded alcohol, and immersed in 0.3% hydrogen peroxide in methanol for 10 min to block endogenous peroxidase activity. Heat-induced antigen retrieval (HIAR) was performed in an electric pressure cooker for 5 min in 10 mM citrate buffer (pH 6.0) for p53 and 10 mM Tris base containing 1 mM ethylenediaminetetraacetic acid (pH 9.0) for OCT1. After HIAR, the sections were cooled to room temperature (RT) for 25 min. The sections were rinsed with 50 mM Tris-buffered saline (TBS, pH 7.6) and incubated with mouse monoclonal anti-p53 (1:100 dilution, clone DO-7; Dako, Glostrup, Denmark) or mouse monoclonal anti-OCT1 (1:2000 dilution, clone 2C5; Novus Biologicals, Centennial, CO, USA) primary antibodies overnight at RT. After rinsing with TBS, sections for p53 staining were incubated with anti-mouse horseradish peroxidase polymer (Histofine Simple Stain MAX-PO; Nichirei Bioscience, Tokyo, Japan) for 1 h at RT. Sections for OCT1 staining were incubated with post-primary block for 30 min at RT, rinsed with TBS, and then incubated with Novolink polymer (Novolink Polymer Detection Systems; Leica Microsystems, Wetzlar, Germany) for 30 min at RT. Sections were then rinsed again with TBS, and the reaction products were developed with a diaminobenzidine solution (Dako). Finally, sections were lightly counterstained with Mayer’s hematoxylin, dehydrated in graded alcohol, cleared in xylene, and coverslipped. Positive controls were sections of colon cancer for p53 and normal liver for OCT1. The primary antibody was replaced with TBS containing 1% bovine serum albumin to provide a negative control.

### Assessment of immunostaining results

The immunostained sections were reviewed by three investigators (MI, TD, and SK) who were blinded to the clinical data. If a disagreement in the scores occurred, the slides were reviewed again, and a consensus was reached. Nuclear staining of p53 was considered positive. Mutation of *TP53* leads to excessive accumulation or loss of p53 protein, and thus, both positive staining in the majority of tumor cells and completely negative staining could be associated with *TP53* mutation [25]. Therefore, we considered that positive staining in ≤ 70% of tumor cells was wild-type expression of p53 (p53^WT-ex^), and positive staining in > 70% of tumor cells or completely negative staining was mutant-type expression of p53 (p53^MT-ex^) [26].

Membranous staining of OCT1 was considered positive. OCT1 expression levels were assessed according to the intensity and percentage of the positive tumor cells. The staining intensity was graded as follows: score 0 (negative), score 1 (weak), score 2 (moderate), and score 3 (strong). The percentage of positive tumor cells was classified as follows: score 0 (none), score 1 (1%–10%), score 2 (11%–50%), and score 3 (> 50%). Furthermore, the immunoreactive score was calculated by adding the intensity score to the percentage score (0–6). Tumors with an immunoreactive score of 0–3 were designated as “low” (OCT1^Low^) and tumors with an immunoreactive score of 4–6 as “high” (OCT1^High^).

### Statistical analysis

Correlations between two variables were estimated using Fisher’s exact test. Multivariate analysis using a logistic regression model was performed to identify independent predictors of histological response. Parameters with a *P*-value of < 0.2 in the univariate analysis were included in the multivariate analysis. *P* < 0.05 was considered statistically significant. All statistical analyses were performed using SPSS Statistics 28 software (IBM Corp., Armonk, NY, USA).

## Results

### Patients’ characteristics

The pretreatment characteristics of patients and tumors are provided in Table 1. This study included 51 men and 15 women with a median age of 67 years (range, 38–79 years). The primary tumor location was the upper thoracic esophagus in eight patients, middle thoracic esophagus in 38 patients, lower thoracic esophagus in 18 patients, and abdominal esophagus in two patients. Regarding histological type, 12 tumors were well differentiated, 42 were moderately differentiated, 7 were poorly differentiated, and 5 were basaloid squamous cell carcinoma. The clinical T factors were cT1 in 2 patients, cT2 in 20 patients, cT3 in 32 patients, and cT4 in 12 patients. Forty-nine patients had lymph node metastasis (clinical N factors were cN1 in 21 patients, cN2 in 19 patients, cN3 in 8 patients, and cN4 in 1 patient). Eighteen patients had cStage II disease, 32 patients had cStage III, and 16 patients had cStage IVa.

**Table 1:** Pretreatment characteristics of patients and tumors

Parameter		Total *n* = 66	CF group *n* = 47	DCF group *n* = 19	*P*-value
Age (years)	≥ 65	39	30 (76.9%)	9 (23.1%)	0.273
	< 65	27	17 (63.0)	10 (37.0)	
Sex	Male	51	38 (74.5)	13 (25.5)	0.335
	Female	15	9 (60.0)	6 (40.0)	
Tumor location	Upper/middle	46	33 (71.7)	13 (28.3)	1.000
	Lower/abdominal	20	14 (70.0)	6 (30.0)	
Histological type	Well/moderate	54	37 (68.5)	17 (31.5)	0.484
	Poor/basaloid	12	10 (83.3)	2 (16.7)	
Clinical T factor	cT1/cT2	22	20 (90.9)	2 (9.1)	0.019*
	cT3/cT4	44	27 (61.4)	17 (38.6)	
Clinical N factor	cN0	17	14 (82.4)	3 (17.6)	0.354
	cN1–cN4	49	33 (67.3)	16 (32.7)	
Clinical stage	II	18	18 (100)	0 (0)	< 0.001*
	III/IVa	48	29 (60.4)	19 (39.6)	

*CF* cisplatin/5-fluorouracil, *DCF* docetaxel/cisplatin/5-fluorouracil

*Statistically significant

Forty-seven patients received preoperative chemotherapy with the CF regimen, and the remaining 19 patients received the DCF regimen. Patients with higher advanced cT and cStage were more frequently included in the DCF group than in the CF group (*P* = 0.019 and *P* < 0.001, respectively). No significant differences in other characteristics were detected between the CF and DCF groups.

### Expression of p53 and OCT1

**Fig. 1:**
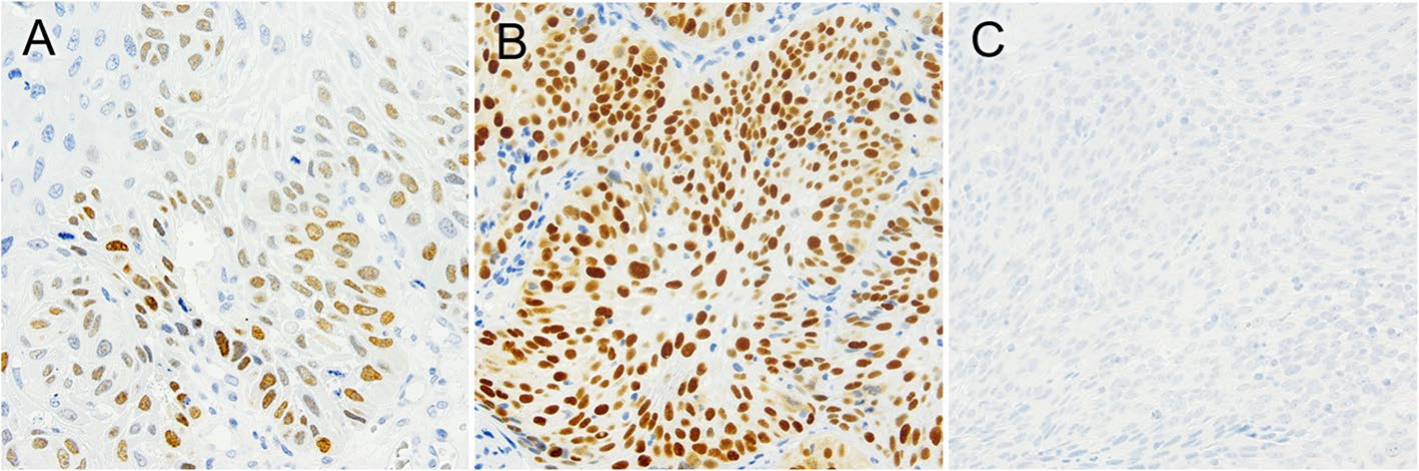
Representative immunostaining patterns of p53 in pretreatment biopsy specimens from ESCC patients. **A** Wild-type expression of p53 with heterogeneous staining, **B** mutant-type expression of p53 with homogeneous (diffuse) staining, and **C** mutant-type expression of p53 with completely negative staining. p53 staining was observed in the nuclei of tumor cells (**A**, **B**)

**Fig. 2:**
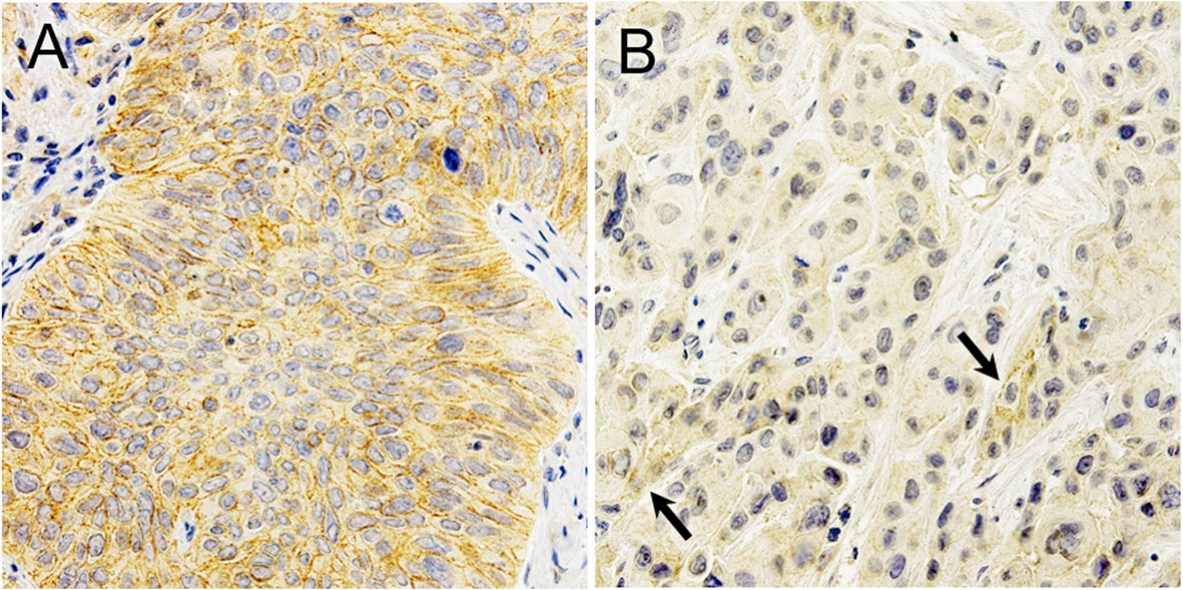
Representative immunostaining patterns of OCT1 in pretreatment biopsy specimens from ESCC patients. **A** High expression of OCT1, with a large number of positive tumor cells, and **B** low expression of OCT1, with only a few positive tumor cells (arrows). OCT1 staining was observed in the cell membranes of tumor cells

Representative staining patterns of p53 and OCT1 in pretreatment biopsy specimens are shown in Figs. 1 and 2, respectively. p53 was expressed in the nucleus, and OCT1 showed membranous staining. p53^WT-ex^ was observed in 27 (40.9%) of 66 tumors, and the remaining 39 (59.1%) tumors were p53^MT-ex^ (28 tumors had positive staining in > 70% of tumor cells, and 11 tumors showed completely negative staining). OCT1^High^ and OCT1^Low^ were observed in 30 (45.5%) and 36 (54.5%) tumors, respectively. No significant correlation of expression status was observed between p53 and OCT1 (*r* = –0.079; *P* = 0.530). p53-positive cells were scattered in the basal layer of nonneoplastic esophageal epithelia. Epithelial cells in the middle and basal layers of nonneoplastic esophageal epithelia showed weak or negative staining of OCT1. In the negative control slides, no positive reaction was observed.

### Correlation between expression status of p53 or OCT1 and clinicopathological characteristics

Table 2 shows the correlation between the expression status of p53 or OCT1 in pretreatment biopsy specimens and clinicopathological characteristics. No significant correlation was found between the expression status of p53 and OCT1 and the clinicopathological parameters of the patients.

**Table 2:** Correlation of p53 and OCT1 expression with clinicopathological parameters

Parameter		*n*	p53^WT-ex^	p53^MT-ex^	*P*-value	OCT1^High^	OCT1^Low^	*P*-value
Age (years)	≥ 65	39	15 (38.5%)	24 (61.5%)	0.799	17 (43.6%)	22 (56.4%)	0.803
	< 65	27	12 (44.4)	15 (55.6)		13 (48.1)	14 (51.9)	
Sex	Male	51	20 (39.2)	31 (60.8)	0.766	24 (47.1)	27 (52.9)	0.770
	Female	15	7 (46.7)	8 (53.3)		6 (40.0)	9 (60.0)	
Tumor location	Upper/middle	46	18 (39.1)	28 (60.9)	0.786	19 (41.3)	27 (58.7)	0.421
	Lower/abdominal	20	9 (45.0)	11 (55.0)		11 (55.0)	9 (45.0)	
Histological type	Well/moderate	54	21 (38.9)	33 (61.1)	0.528	25 (46.3)	29 (53.7)	1.000
	Poor/basaloid	12	6 (50.0)	6 (50.0)		5 (41.7)	7 (58.3)	
Clinical T factor	cT1/cT2	22	7 (31.8)	15 (68.2)	0.426	12 (54.5)	10 (46.5)	0.310
	cT3/cT4	44	20 (45.5)	24 (54.5)		18 (40.9)	26 (59.1)	
Clinical N factor	cN0	17	7 (41.2)	10 (58.8)	1.000	10 (58.8)	7 (41.2)	0.261
	cN1–cN4	49	20 (40.8)	29 (59.2)		20 (40.8)	29 (59.2)	
Clinical stage	II	18	8 (44.4)	10 (55.6)	0.783	9 (50.0)	9 (50.0)	0.783
	III/IVa	48	19 (39.6)	29 (60.4)		21 (43.8)	27 (56.2)	
Chemotherapy regimen	CF	47	20 (42.6)	27 (57.4)	0.785	23 (48.9)	24 (51.1)	0.423
	DCF	19	7 (36.8)	12 (63.2)		7 (36.8)	12 (64.2)	

*CF* cisplatin/5-fluorouracil, *DCF* docetaxel/cisplatin/5-fluorouracil, *OCT1* organic cation transporter 1

### Correlation of p53 and OCT1 expression status with histological response in the entire cohort

**Fig. 3:**
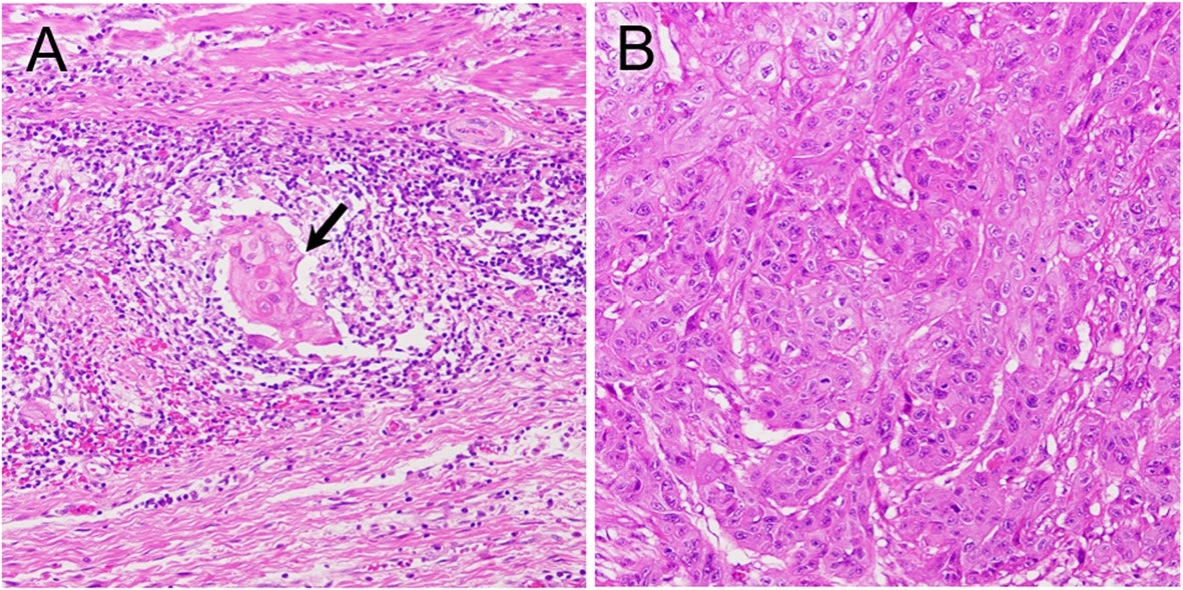
H&E findings of posttreatment resection materials from ESCC patients. In a tumor with a histological grade 2 response (**A**), a few tumor cells (arrow) remained with remarkable infiltration of inflammatory cells. However, in a tumor with grade 0 (**B**), no histological changes were discernible

**Fig. 4:**
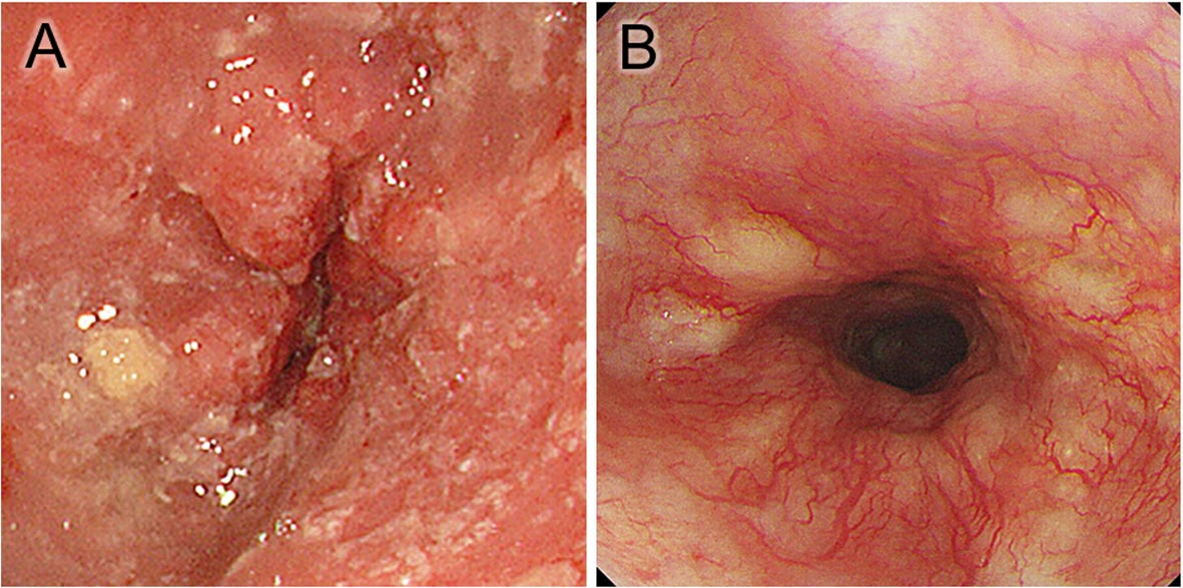
Endoscopic findings of a tumor with a histological grade 2 response from an ESCC patient. Pretreatment endoscopy showed an irregularly protruded lesion (**A**). After preoperative chemotherapy, the tumor had almost disappeared (**B**)

A histological complete response (grade 3) in posttreatment resection materials was achieved in 5 tumors. Grade 2 was achieved in 5 tumors (Fig. 3A), 9 tumors were grade 1b, 45 tumors were grade 1a, and 4 tumors were grade 0 (Fig. 3B). The endoscopic findings of a tumor with a histological grade 2 response are shown in Fig. 4. Table 3 shows the correlation of clinicopathological parameters or the expression status of p53 and OCT1 in the pretreatment biopsy specimens with histological response in the entire cohort. In the univariate analysis, no correlation with histological response was detected for age, sex, tumor location, histological type, clinical T factor, clinical N factor, clinical stage, or chemotherapy regimen, whereas p53^MT-ex^ was significantly correlated with poor response. Indeed, 33 (84.6%) of the 39 tumors with p53^MT-ex^ and 16 (59.3%) of the 27 tumors with p53^WT-ex^ showed a poor response (*P* = 0.026). Interestingly, all 11 tumors with completely negative staining showed a poor response. The sensitivity of p53 expression for predicting poor response was 67.3% (33 tumors with p53^MT-ex^ out of the 49 poorly responsive tumors), and the specificity was 64.7% (11 tumors with p53^WT-ex^ out of the 17 responsive tumors). The accuracy was 66.7%, 44 (33 poorly responsive tumors with p53^MT-ex^ and 11 responsive tumors with p53^WT-ex^) out of the 66 tumors. OCT1^Low^ was marginally correlated with poor response (*P* = 0.091).

**Table 3:** Correlation of p53 and OCT1 expression with histological response in the entire cohort

Parameter		*n*	Histological response		*P*-value
			Poor	Good	
Age (years)	≥ 65	39	29 (74.4%)	10 (25.6%)	1.000
	< 65	27	20 (74.1)	7 (25.9)	
Sex	Male	51	40 (78.4)	11 (21.6)	0.185
	Female	15	9 (60.0)	6 (40.0)	
Tumor location	Upper/middle	46	36 (78.3)	10 (21.7)	0.359
	Lower/abdominal	20	13 (65.0)	7 (35.0)	
Histological type	Well/moderate	54	42 (77.8)	12 (22.2)	0.271
	Poor/basaloid	12	7 (58.3)	5 (41.7)	
Clinical T factor	cT1/cT2	22	17 (77.3)	5 (22.7)	0.773
	cT3/cT4	44	32 (72.7)	12 (27.3)	
Clinical N factor	cN0	17	14 (82.4)	3 (17.6)	0.525
	cN1–cN4	49	35 (71.4)	14 (28.6)	
Clinical stage	II	18	15 (83.3)	3 (16.7)	0.361
	III/IVa	48	34 (70.8)	14 (29.2)	
Chemotherapy regimen	CF	47	36 (76.6)	11 (23.4)	0.542
	DCF	19	13 (68.4)	6 (31.6)	
p53 expression	Wild type	27	16 (59.3)	11(40.7)	0.026*
	Mutant type	39	33 (84.6)	6 (15.4)	
OCT1 expression	High	30	19 (71.4)	11 (28.6)	0.091
	Low	36	30 (83.3)	6 (16.7)	
p53/OCT1 expression	Both p53^WT-ex^ and OCT1^High^	11	3 (27.3)	8 (72.7)	< 0.001*
	Either p53^MT-ex^ or OCT1^Low^	55	46 (83.6)	9 (16.4)	

*CF* cisplatin/5-fluorouracil, *DCF* docetaxel/cisplatin/5-fluorouracil, *OCT1* organic cation transporter 1

*Statistically significant

Multiple biological processes are involved in tumor response to chemotherapy, and therefore, we also investigated the combination of p53 and OCT1 expression. Poor response was observed in 46 (83.6%) of the 55 tumors with either p53^MT-ex^ or OCT1^Low^ expression and 3 (27.3%) of the 11 tumors with both p53^WT-ex^ and OCT1^High^ (*P* < 0.001: a higher level of statistical significance compared with p53 alone). The sensitivity of combined p53/OCT1 for predicting poor response was 93.9% (46 tumors with either p53^MT-ex^ or OCT1^Low^ out of the 49 poorly responsive tumors), and the specificity was 47.1% (8 tumors with both p53^WT-ex^ and OCT1^High^ out of the 17 responsive tumors). The accuracy was 81.8%: 54 (46 poorly responsive tumors with either p53^MT-ex^ or OCT1^Low^ and 8 responsive tumors with both p53^WT-ex^ and OCT1^High^) out of the 66 tumors. Combined p53/OCT1 had higher sensitivity and accuracy but lower specificity compared with p53 alone.

### Multivariate analysis of predictors of histological response in the entire cohort

p53 expression and combined p53/OCT1 were not included in the same multivariate model to avoid multicollinearity (Table 4). Patient sex and p53 and OCT1 expression were included in Model 1, and patient sex and combined p53/OCT1 was included in Model 2. p53 expression (odds ratio [OR], 2.16; 95% confidence interval [CI], 1.15–4.07; *P* = 0.017), OCT1 (*OR*, 2.02; 95% *CI*, 1.06–3.85; *P* = 0.032), and combined p53/OCT1 (*OR*, 3.79; 95% *CI*, 1.75–8.21; *P* < 0.001; the highest level of statistical significance) were significant predictors of histological response. However, patient sex was not identified as an independent predictor of histological response.

**Table 4:** Multivariate analysis of predictors of histological response in the entire cohort

Parameters		*OR* (95% *CI*)	*P*-value
Model 1			
Sex	Male vs. female	1.67 (0.84–3.32)	0.147
p53 expression	Wild type vs. mutant type	2.16 (1.15–4.07)	0.017*
OCT1 expression	High vs. low	2.02 (1.06–3.85)	0.032*
Model 2			
Sex	Male vs. female	1.65 (0.82–3.33)	0.165
p53/OCT1 expression	Both p53^WT-ex^ and OCT1^High^ vs. either p53^MT-ex^ or OCT1^Low^	3.79 (1.75–8.21)	< 0.001*

*CI* confidence interval, *OCT1* organic cation transporter 1, *OR* odds ratio

*Statistically significant

### Correlation of the expression status of p53 and OCT1 with histological response according to chemotherapy regimen

We attempted to perform further analyses in which CF and DCF groups were assessed separately. In the CF group (*n* = 47), p53 (*P* = 0.165) and OCT1 (*P* = 0.318) expression did not correlate with histological response (Table 5). However, when evaluated according to the combination of p53 and OCT1 data, a poor response was observed in 33 (84.6%) of the 39 tumors with either p53^MT-ex^ or OCT1^Low^ and 3 (37.5%) of the 8 tumors with both p53^WT-ex^ and OCT1^High^ (*P* = 0.011). No other parameters were correlated with histological response. Because the parameters with *P*<0 .2 were p53 alone and combined p53/OCT1, we did not perform the multivariate analysis.

**Table 5:** Correlation of p53 and OCT1 expression with histological response in the CF group

Parameter		*n*	Histological response		*P*-value
			Poor	Good	
Age (years)	≥ 65	30	22 (73.3)	8 (26.7)	0.722
	< 65	17	14 (82.4)	3 (17.6)	
Sex	Male	38	30 (78.9)	8 (21.1)	0.419
	Female	9	6 (66.7)	3 (33.3)	
Tumor location	Upper/middle	33	27 (81.8)	6 (18.2)	0.263
	Lower/abdominal	14	9 (64.3)	5 (35.7)	
Histological type	Well/moderate	37	30 (81.1)	7 (18.9)	0.213
	Poor/basaloid	10	6 (60.0)	4 (40.0)	
Clinical T factor	cT1/cT2	20	17 (85.0)	3 (15.0)	0.481
	cT3/cT4	27	20 (74.1)	7 (25.9)	
Clinical N factor	cN0	14	12 (85.7)	2 (14.3)	0.464
	cN1/cN2/cN3	33	24 (72.7)	9 (27.3)	
Clinical stage	II	18	15 (83.3)	3 (16.7)	0.492
	III/IVa	29	21 (72.4)	8 (27.6)	
p53 expression	Wild type	20	13 (65.0)	7 (35.0)	0.165
	Mutant type	27	23 (85.2)	4 (14.8)	
OCT1 expression	High	23	16 (69.6)	7 (30.4)	0.318
	Low	24	20 (83.3)	4 (16.7)	
p53/OCT1 expression	Both p53^WT-ex^ and OCT1^High^	8	3 (37.5)	5 (62.5)	0.011*
	Either p53^MT-ex^ or OCT1^Low^	39	33 (84.6)	6 (15.4)	

*CF* cisplatin/5-fluorouracil, *OCT1* organic cation transporter 1

*Statistically significant

Combined p53/OCT1 was also the only significant parameter in the DCF group, although the values should only be used as references because of the small number of cases (*n* = 19): a poor response was observed in 13 (81.3%) of the 16 tumors with either p53^MT-ex^ or OCT1^Low^ and in none of the 3 tumors with both p53^WT-ex^ and OCT1^High^ (*P* = 0.021).

## Discussion

Although preoperative chemotherapy with the CF-based (CF and DCF) regimens has been established as an effective therapy for ESCC patients, the treatment outcomes remain unsatisfactory [3–5]. Therefore, the identification of predictive biomarkers is important for selecting subgroups of patients who will be responsive or unresponsive to preoperative chemotherapy. In this study, we evaluated whether response to preoperative chemotherapy for ESCC could be predicted by immunohistochemical staining of p53 and OCT1 in pretreatment biopsy specimens.

We found that p53^MT-ex^, which is used as a surrogate marker of *TP53* mutation, was significantly correlated with poor response in the univariate (*P* = 0.026) and multivariate (*P* = 0.017) analyses of the entire cohort. The value of p53 protein expression for predicting response to preoperative chemotherapy in ESCC remains controversial. Several studies suggested a higher response rate of p53-negative tumors to preoperative chemotherapy than p53-positive tumors [24, 27, 28], while others reported no significant difference in responsiveness between p53-positive and p53-negative tumors [10, 29–31]. Conversely, data from *TP53* mutation analyses indicated the correlation of *TP53* mutation and poor response to preoperative chemotherapy [10, 32].

Generally, it is accepted that wild-type p53 has a short half-life, which makes it immunohistochemically undetectable [33]. In contrast, mutation of *TP53* increases the half-life of p53 protein, leading to nuclear accumulation of mutant-type p53, which is immunohistochemically detectable. However, p53-positive staining does not consistently indicate the presence of *TP53* mutation, and negative staining does not represent the absence of abnormalities in *TP53* [34, 35]. Indeed, previous studies demonstrated that the concordance rate between p53 protein expression and *TP53* mutation in esophageal cancer was approximately 50% when staining of > 10% of tumor cells was regarded as positive [11, 12].

We would like to emphasize that the expression pattern of p53 protein, that is*,* the proportion of p53-positive cells, is of great importance in discussing the correlation between p53 protein expression and *TP53* gene mutation [25, 33, 35]. A diffuse (homogeneous) expression pattern could correlate with *TP53* mutation. Furthermore, completely negative staining should also be interpreted as being consistent with mutant-type p53 because the presence of null mutations, such as frameshift or nonsense mutations, could result in the complete absence of immunoexpression due to the abolishment of protein production [7, 25]. Conversely, a focal or heterogeneous expression pattern does not seem to be associated with obvious abnormalities of *TP53*. This may be explained by a non-mutational mechanism for p53 protein accumulation, such as inactivation of the p53 degradation process and spontaneous genetic errors [33, 34]. Therefore, in our study, p53 expression was classified into two groups: p53^MT-ex^ (positive staining in > 70% of tumor cells or completely negative staining) versus p53^WT-ex^ (positive staining in ≤ 70% of tumor cells) [26]. In previous studies, however, different criteria for determining p53 immunopositivity were provided. Several studies defined p53 positivity as nuclear staining in > 10% of the tumor cells [10, 24, 28] but others as nuclear staining in > 25% [30] or > 50% [31] of the tumor cells. In these criteria, however, completely negative staining is judged as consistent with wild-type p53, which may be an important cause of discordance between the p53 immunopositivity and the genetic abnormality. Importantly, in our study, completely negative staining was noted in 11 tumors, all of which showed a poor response. Taken together, a significant correlation between p53^MT-ex^ and poor response to preoperative chemotherapy in the univariate analysis of our series may reflect the presence of mutant-type p53 that leads to the resistance to chemotherapy-induced apoptosis. However, our results must be confirmed by further studies using a *TP53* mutational analysis, such as single-strand conformational polymorphism and direct sequencing.

Different regimens, doses, and courses of preoperative chemotherapy, different evaluation criteria of response to preoperative chemotherapy, and different staining protocols and sensitivity of p53 immunohistochemistry may result in conflicting results. Thus, standardization is necessary to obtain an accurate assessment of the significance of p53 expression in response to preoperative chemotherapy in ESCC.

An in vitro study demonstrated that *OCT1* silencing impaired the cytotoxic effect of CDDP in ESCC cells, and that the level of *OCT1* mRNA expression was downregulated in CDDP-resistant cells compared with the parental cells [11]. However, the movement of CDDP across membranes is greatly influenced not only by OCT1 but also by OCT2 and copper transporter 1 (CTR1) [15–18]. Although we investigated OCT2 and CTR1 as a preliminary examination, high expression of OCT2 and CTR1 was observed in only 6 and 2 of the 66 tumors, respectively (data not shown). These findings suggest that OCT1 may be a major CDDP-uptake transporter in ESCC. However, to our knowledge, no previous studies have investigated the predictive significance of OCT1 expression in response to preoperative chemotherapy in clinical ESCC samples. Therefore, in the present study, we assessed whether OCT1 expression in pretreatment biopsy specimens was significantly correlated with histological response to preoperative chemotherapy in ESCC. The correlation of OCT1 with histological response was marginal in the univariate analysis (*P* = 0.091) but significant in the multivariate analysis (*P* = 0.032). This suggests that the effects of preoperative chemotherapy for ESCC are difficult to predict by OCT1 alone because of its low levels of statistical significance.

Tumor response to chemotherapy is a multifactorial event, and therefore, a combination of biomarkers seems reasonable for accurately and/or reliably predicting the effectiveness of preoperative chemotherapy in ESCC. In this study, when p53 and OCT1 expression data were combined, either p53^MT-ex^ or OCT1^Low^ was a significant predictor of poor response in univariate and multivariate analyses of the entire cohort (univariate, *P* < 0.001; multivariate, *P* < 0.001), with higher levels of statistical significance compared with p53 alone, and in the univariate analysis of the CF group (*P* = 0.011) and the DCF group (*P* = 0.021). The poor responsiveness of tumors with either p53^MT-ex^ or OCT1^Low^ suggests that the restricted function of p53 and OCT1 may determine the decreased response through impairing the intracellular uptake of CDDP and/or drug-induced apoptosis. Conversely, combined p53/OCT1 had high sensitivity and accuracy, whereas the specificity was insufficient. The identification of additional biomarkers is required to improve the predictive specificity.

This retrospective study had several limitations: (1) the chemotherapy regimen included CF and DCF, (2) the patients were at different clinical stages, (3) this study was performed at a single institution and lacked *TP53* gene analysis, and (4) the sample size was small, resulting in low statistical power. Therefore, our results are still exploratory. They should be interpreted cautiously and need to be confirmed in prospective studies of large patient cohorts at the same stage of disease and in patients receiving the same chemotherapy regimen.

## Conclusions

This study suggests that either p53^MT-ex^ or OCT1^Low^ in pretreatment biopsy specimens may be a potential predictor of poor response to preoperative chemotherapy with the CF-based regimens in ESCC. Patients with p53^MT-ex^/OCT1^Low^ tumors may be candidates for a more aggressive combination protocol or new drugs. However, several issues, particularly the insufficient specificity, need to be resolved.

## Data Availability

The data that support the findings of this study are available from the corresponding author upon reasonable request.

## Abbreviations

CDDP: Cisplatin
CF: Cisplatin/5-fluorouracil
CI: Confidence interval
CTR1: Copper transporter 1
DCF: Docetaxel/cisplatin/5-fluorouracil
ESCC: Esophageal squamous cell carcinoma
5-FU: 5-fluorouracil
HIAR: Heat-induced antigen retrieval
OCT1: Organic cation transporter 1
OCT1^High^: High expression of organic cation transporter 1
OCT1^Low^: Low expression of organic cation transporter 1
OR: Odds ratio
p53^MT-ex^: Mutant-type expression pattern of p53
p53^WT-ex^: Wild-type expression pattern of p53
RT: Room temperature
SLC: Solute carrier
TBS: Tris-buffered saline
